# Secure and efficient routing on nodes, edges, and arcs of simple‐graphs and of multi‐graphs

**DOI:** 10.1002/net.21993

**Published:** 2020-09-25

**Authors:** Georg E. A. Fröhlich, Karl F. Doerner, Margaretha Gansterer

**Affiliations:** ^1^ Department of Business Decisions and Analytics University of Vienna Vienna Austria; ^2^ Data Science University of Vienna Vienna Austria; ^3^ Department for Operations Management and Logistics University of Klagenfurt Klagenfurt Austria

**Keywords:** adaptive large neighborhood search, inconsistency, multi‐objective optimization, vehicle routing, epsilon box splitting heuristic, multi‐graph

## Abstract

Many security companies offer patrolling services, such that guards inspect facilities or streets on a regular basis. Patrolling routes should be cost efficient, but the inspection patterns should not be predictable for offenders. We introduce this setting as a multi‐objective periodic mixed capacitated general routing problem with objectives being cost minimization and route inconsistency maximization. The problem is transformed into an asymmetric capacitated vehicle routing problem, on both a simple‐graph and a multi‐graph; and three multi‐objective frameworks using adaptive large neighborhood search are implemented to solve it. As tests with both artificial and real‐world instances show that some frameworks perform better for some indicators, a hybrid search procedure, combining two of them, is developed and benchmarked against the individual solution methods. Generally, results indicate that considering more than one shortest path between nodes, can significantly increase solution quality for smaller instances, but is quickly becoming a detriment for larger instances.

## 1 INTRODUCTION

Security companies offer a range of services to protect objects (e.g., buildings, cash, other valuables) and people (e.g., personal protection, patrolling services). To plan for and assign their personnel efficiently, these companies must resolve challenging routing problems. Attacks theoretically could take place anywhere during the route; either at a point being serviced, or while traversing between these point. Sometimes service points might make good targets to intercept transports, but not always; military facilities, central banks, or locations with a high proximity to police stations are well‐guarded and, therefore, not attractive targets. In those cases, attacking vehicles while they are moving between them might be the more rational choice. To complicate this for robbers, times of traversing the street segments or the street segments themselves can be varied. We would argue that varying times alone is not sufficient, since this can cause street segments that are always traversed, but only at different times, allowing for easy ambushes. Instead more emphasis should be put on how often street segments are used in combination by how often the sequence of services show similarities.

Both visited nodes and traversed edges are relevant for solving this problem, which accordingly reflects a type of the general routing problem (cf. [32]) and the node, edge, and arc routing problem introduced in [36].

We propose to model this problem as a multi‐objective periodic mixed capacitated general routing problem (MO‐P‐MCGRP), in which nodes, edges, and arcs must be visited regularly but with inconsistent routes to ensure the unpredictability of visiting or service patterns. This problem is NP‐hard, since it is a generalization of the capacitated vehicle routing problem (CVRP). Its two objectives are to minimize the cost and to maximize the route inconsistency. Because maximizing route inconsistency and minimizing route consistency are interchangeable, we model the problem with consistency; a multi‐objective problem becomes easier to represent if all objectives must be either maximized or minimized. But we will refer to maximizing inconsistency, while measuring route consistency by how often an edge/arc gets traversed and how often pairs of required nodes, edges, and arcs are serviced directly after one another. Since for most street segments ambushes could be set on either side with nearly the same effect, we deemed the direction in which edges are traversed irrelevant for the route consistency.

**FIGURE 1:**
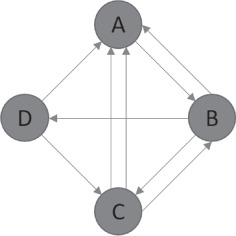
Example of multi‐graph with two arcs going from node C to node A [Color figure can be viewed at wileyonlinelibrary.com]

**FIGURE 2:**
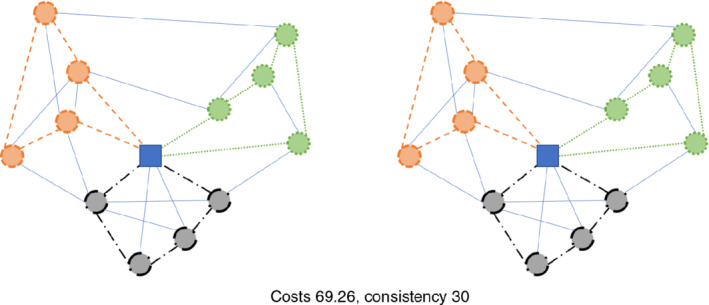
Example of a node‐based routing problem (3 tours) with low inconsistency over 2 periods (period 1 left; period 2 right) [Color figure can be viewed at wileyonlinelibrary.com]

**FIGURE 3:**
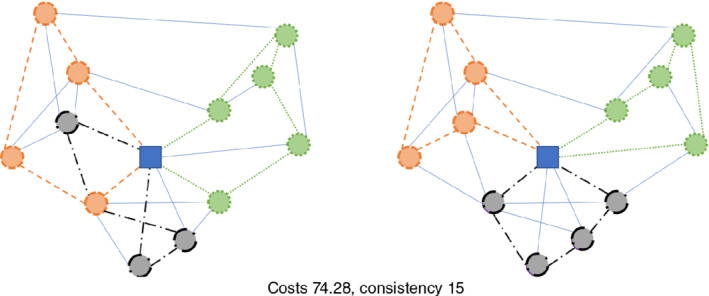
Example of a node‐based routing problem (3 tours) with increased inconsistency (compared to Figure 2) over 2 periods (period 1 left; period 2 right) [Color figure can be viewed at wileyonlinelibrary.com]

For solving the problem, we also use a conversion into a multi‐graph, where several arcs with the same orientation can exist between two nodes. Although MCGRPs often get converted into simple‐graphs, this conversion can lead to a loss in solution quality, whereas this issue can be mitigated with the use of a multi‐graph. Figure 1 depicts a simple multi‐graph with two arcs from node *C* to *A*. Figures 2 and 3 both depict solutions for two periods, in which only the nodes need to be serviced. Figure 2 shows a very poor solution for inconsistency in that the sequence of nodes and used edges is the same in both periods. The solution in Figure 3 is 7.25% worse for costs but 50% better in terms of inconsistency, because fewer edges are repeated, and the sequence of nodes gets changed.

Although any multi‐objective problem can be converted into a single‐objective problem by weighting the individual objectives and adding them, objectives that differ in their nature might be more difficult to combine, such as a monetary objective (costs) and a non‐monetary objective (inconsistency). Therefore, it might be more practical to solve the multi‐objective problem by finding the Pareto set, or a set of non‐dominated solutions, and then suggesting rules for choosing from this set. Furthermore, decision makers might not want to share their precise preferences regarding objectives a priori (cf. [15]), in which case the appropriate conversion of a multi‐objective problem into a single‐objective more difficult.

Dominance in the context of multi‐objective problems arises in the following way: Assume two solutions *S*_1_ = {*s*_11_, … , *s*_1*n*_} and *S*_2_ = {*s*_21_, … , *s*_2*n*_}, where *s*_*ij*_ refers to the individual objective values that must be minimized. Solution *S*_1_ dominates *S*_2_ if the following holds: *s*_1*j*_ ≤ *s*_2*j*_ for all objectives and *s*_1*j*_ < *s*_2*j*_ for at least one objective. A solution not being dominated by any solution, is part of the Pareto set. Solutions of the Pareto set can furthermore be classified into supported and non‐supported solutions. For this, the relevant criterion is whether there exists a combination of weights for a weighted sum of objectives such that the solution is optimal. For example, assume {1, 0}, {0.51, 0.51}, and {0, 1} are non‐dominated solutions for a problem; {1, 0} and {0, 1} are supported, but {0.51, 0.51} is not. The conversion of a multi‐objective problem into a single‐objective problem via weighing the objectives would therefore only allow supported solutions of the Pareto set to emerge and thus might exclude or ignore some promising trade‐off solutions.

In our effort to contribute to this field of research, we:

- Introduce our proposed MO‐P‐MCGRP as a means to minimize cost and consistency (maximize route inconsistency) and mathematically formulate its conversion to a node‐based formulation on a multi‐graph.
- Develop an adaptive large neighborhood search (ALNS)‐based algorithm for the single‐objective problem and embed it into three multi‐objective frameworks.
- Analyze performance using artificial and real‐world instances for simple‐ and multi‐graphs. All instances are made publicly available on the BDA homepage [3].
- Deliver insights on the trade‐off between cost and inconsistency according to the real‐world instances.
- Show that the solutions benefit from a higher degree of freedom provided by the multi‐graphs and also specify how the gain may differ with target performance indicators and problem sizes.

In Section 2 we discuss related literature. Then in Section 3, we provide a problem description for the MO‐P‐MCGRP and the conversion of the MO‐P‐MCGRP, followed by the ALNS procedure and its incorporation into multi‐objective frameworks in Section 4. After we present the computational study in Section 5, we summarize the findings and their implications in Section 6. Additionally, the detailed mathematical model of the conversion of the MO‐P‐MCGRP can be found in the Appendix.

## 2 LITERATURE REVIEW

Reflecting our focus on a MO‐P‐MCGRP and security aspects, we discuss research contributions pertaining to (1) routing with security aspects, (2) MCGRP, (3) multi‐graphs, and (4) multi‐objective problems.

A safe and secure routing problem arises when cash or patrol guards must be transported or scheduled. In cash‐in‐transit operations and patrolling, unpredictability is a crucial issue that usually is addressed by paths that are inconsistent in their routes or times. Route inconsistency requires the adoption of different edges and arcs; time inconsistency implies performing the service at different points in time. We focus on route inconsistency. The m‐peripatetic vehicle routing problem (m‐PVRP), which aims at route inconsistency, is analyzed in [31], where a hybrid tabu search is used. In [50], the authors consider a security problem with three service types and assume that some nodes must be visited only once, while others are served multiple times. A third set of services refers to alarms, which are not known a priori. The authors use a two‐stage approach, such that they first cluster the alarms according to a capacitated p‐median algorithm and then solve the routing and scheduling problems with an adapted version of alternate k‐exchange reduction, which builds a set of initial solutions and the improves them via local search [51]. Cost‐effective routing including security aspects is considered in [52], where the problem is split into subproblems, solved with CPLEX. A solution is considered secure if not too many routes within it are similar, defined by the number of demand points visited at similar points in time. In studies of a periodic VRP with time windows and time spread constraints, authors use an iterated granular tabu search [21] or a multi‐start iterated local search [30]. Visiting a node at a point in time that is too close to a previous visit of the node is deemed insecure. Multiple heuristics, like iterated local search and an ant colony optimization for the risk constrained cash‐in‐transit VRP [41, 42], might involve risk defined as the amount of money being transported at a given point in time. The proposed methods aim to find cost effective solutions, at a risk level below a given threshold. The methods' authors provide several new benchmark instances for their problem. In [43], the authors introduce the multi‐objective risk‐constrained problem with two objectives, namely cost and risk minimization. Risk in their case is the sum product of money and the distance it gets transported. Their multi‐objective optimization method is based on iterated local search. However, they do not focus on generating a Pareto set approximation but instead attempt to integrate the decision maker's preference into the optimization process by providing a subset of appropriate solutions.

The single‐objective MCGRP has been analyzed from several research groups. In [8], the authors use an integer programming model and branch‐and‐cut algorithm to introduce the problem and provide benchmark instances (MGGDB). In [7], a matheuristic is applied to the newly introduced problem. This method consists of a destroy‐and‐repair algorithm for diversification, a variable neighborhood descent for local search, and the aforementioned branch‐and‐cut algorithm. A memetic algorithm for the MCGRP as well as a new set of instances (CBMix) are introduced in [36]. Further instances (BHW and DI‐NEARP) as well as a lower bound procedure are provided in [1]. Most of the currently best known results for these instances have been firstly published in [12], where an adaptive iterated local search is used, and [45], where the multi‐start iterated local search from [35] and the unified hybrid genetic search from [46, 47] are applied. Most of the best lower bounds for BHW, CBMix, and MGGDB are either reproduced or found in [2]. The authors apply a branch‐and‐cut‐and‐price algorithm that combines cut generation and column generation. Multi‐objective versions of the MCGRP are also studied in [19]. The authors focus on cost and route balance, with multiple different measurements. A set‐partitioning model and the box splitting method from [20] are used to solve some of the smaller MGGDB instances. Besides those problems, MCGRPs with some more restrictions are studied in, for example, [10], where a branch‐price‐and cut is applied to a MCGRP with time windows. A MCGRP with turn penalties is presented in [45]. The same problem is investigated in [9], where it is transformed into a VRP and then solved via a memetic algorithm. A MCGRP with stochastic demands is tackled in [6], where a branch‐and‐cut as well as a matheuristic combining local search and branch‐and‐cut are proposed.

Several heuristics convert the MCGRP into a VRP with the shortest paths connecting the nodes of the VRP, but for some routing problems conversion schemes into a simple‐graph are not sufficient. This is pointed out in [16]. The authors note that once multiple attributes are defined on arcs or edges, a transformation of a problem into a standard VRP can lead to the risk of losing optimality and good solutions. They conduct experiments with simple‐ and multi‐graphs and show that improvements can be gained with the latter. In [24], the authors perform some similar experiments for a heterogeneous VRP with limited duration with two arcs between any pair of vertices. They make similar observations as [16]. A multi‐graph for a time dependent alternative VRP, where time windows and travel times depend on the time of the day, is studied in [48]. From the two edges between any pair of nodes, the first has a time‐dependent travel speed distribution, while the second has an, except for peak hours, far longer, but constant travel time. A multi‐graph is also used in [40], where a VRP with arrival time diversification is investigated. The authors assume that customers must be serviced multiple times. The aim is to optimize costs, while having sufficiently different service times at each customer. It is shown that that the increased flexibility due to the multi‐graph enables for better solutions.

For multi‐objective problems several solution approaches are developed. In [29], the authors compare the *ϵ*‐constraint heuristic (ECH) and *ϵ*‐box splitting heuristic (EBSH) while using the hybrid genetic search from [46]. They then compare their method to the greedy randomized adaptive search procedure with advanced starting point introduced in [33] and the multi‐start split‐based path relinking in [23]. However, the results of [29] show that ECH and EBSH are significantly better than the other two. Multi‐directional local search (MDLS) is proposed in [44]. The authors show that they perform comparable to other state‐of‐the‐art methods for multiple problems like the non‐dominated sorting genetic algorithm [13], the strength Pareto evolutionary algorithm 2 [54], the multi‐objective genetic local search [22], and the multi‐objective evolutionary algorithm based on decomposition [53]. MDLS, ECH, and EBSH need an underlying heuristic. For this, we use ALNS, which is a renowned metaheuristic that has been successfully applied to several planning problems (e.g., [17, 34, 39]) including a multi‐graph in [4]. Furthermore, an ALNS is presented in [12], where it is used to generate benchmark results for several of the MCGRP instances. Hence, it is considered a valid approach for our extension.

Compared to the found literature on MCGRP we are, to the best of our knowledge, the first to tackle the MCGRP with the two objectives cost minimization and route inconsistency in a multi‐objective setting. Route inconsistency is measured by the number of times an arc or edge is traversed, excluding service, and by how often required elements are visited in the same order. The former aspect of route inconsistency has similarities to the m‐PVRP (e.g., [31]), since it is based on edges and arcs. Conversions of the MCGRP into a simple‐graph are already performed in [12, 45], but not into a multi‐graph, since their problems are not facing the risk loss of optimality as pointed out in [16] when converting them. Additionally, MDLS, ECH, and EBSH have not been used on a multi‐graph before.

## 3 PROBLEM FORMULATION

### 3.1 Problem description

The MO‐P‐MCGRP can be represented on a graph *G*_*MO* − *P* − *MCGRP*_ = (*N* ∪ *E* ∪ *A*), where *N*, *E*, and *A* are the sets of nodes, edges, and arcs, respectively. Every period, subsets, composed of the *required* nodes *N*_*R*_ ⊆ *N*, edges *E*_*R*_ ⊆ *E*, and arcs *A*_*R*_ ⊆ *A*, must be serviced by a fleet of vehicles that must start and end their routes at the depot, each with a limited capacity. Traveling on edges and arcs causes costs. For the required nodes, edges, and arcs, we consider additional costs for service and demands.

The MO‐P‐MCGRP has two objectives: (1) cost minimization and (2) maximization of route inconsistency (expressed as minimization of route consistency) between different periods.

For route inconsistency, we assume an adapted version of the R‐type distance [28]. For two sequences *p* = (*p*_1_, …, *p*_*n*_) and *q* = (*q*_1_, …, *q*_*m*_), the R‐type distance *d*(*p*, *q*) is the number of times *p*_*i* − 1_ does not immediately follow *p*_*i*_ in *q* for *i* = 1, …, *n* − 1. We adapt this measure so that we can consider inconsistency in regard to nodes, edges, and arcs. We assume that inconsistency diminishes if.

1. An edge or arc is used more than once ignoring the direction, in which edges are traversed. This point does not apply to required edges or arcs (*E*_*R*_, *A*_*R*_), if they are serviced.
2. Two required nodes, edges, or arcs directly follow each other. This point applies even if non‐required nodes, edges, or arcs are traversed in between them.

For this, we determine how often (*x*) every edge and arc is traversed (excluding service) and set the generated consistency *v*_*l*_ of the individual segment *l* to *x* − 1. Similarly, we determine how often (*y*) a specific required node, edge, or arc *i* is serviced directly before servicing a second required node, edge, or arc *j*, and set the generated consistency *v*_*ij*_ to *y* − 1.

### 3.2 Transformation of the Mixed Capacitated General Routing Problem to the Capacitated Vehicle Routing Problem

The MCGRP can be transformed to an asymmetric CVRP on a complete graph *G*_*CVRP*_ = (*V*, *A*) [45]. The union set of required nodes, edges, and arcs (*N*_*R*_, *E*_*R*_, and *A*_*R*_) and the depot *N*_0_ composes a set of nodes *V* ≔ *N*_*R*_ ∪ *E*_*R*_ ∪ *A*_*R*_ ∪ *N*_0_. We use the term point of interest (POI) to refer to any node resulting from this transformation, to differentiate them from the nodes on the original graph. The paths between all POIs in *V* are calculated to create a complete set of arcs *A*. However, POIs that were edges before need special consideration. They can be represented as two sub‐POIs, from which only one must be visited, or there might be multiple shortest paths between them and other POIs, which would result in a multi‐graph.

**FIGURE 4:**
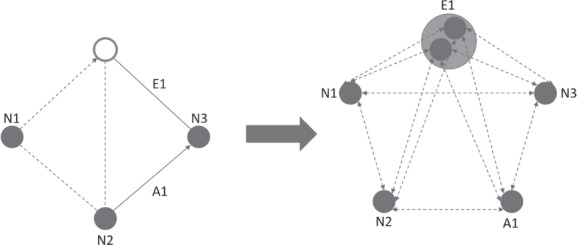
Transformation of a MCGRP to a CVRP. Full circles and lines represent required nodes/edges/arcs. Double‐sided arrows represent two arcs going in opposite directions [Color figure can be viewed at wileyonlinelibrary.com]

**FIGURE 5:**
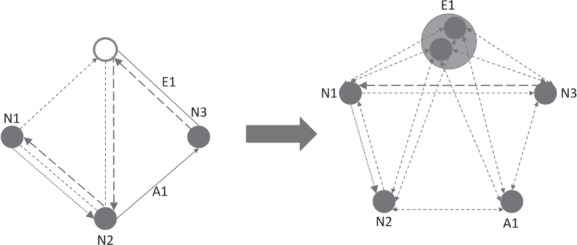
Paths of created CVRP on the original MCGRP. Bold dotted and dashed line in the MCGRP illustrate the paths used for the bold dotted and dashed line in the CVRP [Color figure can be viewed at wileyonlinelibrary.com]

Figure 4 shows such a transformation. The three full nodes, the full edge, and the full arc require service. The arc is converted into a POI, and the edge is converted into one POI with two sub‐POIs, of which only one must be visited. The double‐sided arrows simplify the figure and do not represent edges but instead reflect two arcs going in opposite directions. It is important to note that paths of the conversion often consist out of several edges and arcs from the original problem. This information has to be stored, to calculate route inconsistency. Figure 5 shows the original edges and arcs used for the path from node 3 (N3) to node 1 (N1) and for the path from node 1 (N1) to node 2 (N2).

### 3.3 Transformation of the multi‐objective periodic mixed capacitated general routing problem

The transformation in Section 3.2 has some limitations when being applied in the same manner to the MO‐P‐MCGRP. In the case of the standard MCGRP, the objective is to minimize costs and calculating the shortest paths between the new POIs is sufficient to guarantee that an optimal solution on *G*_CVRP_ is an optimal solution on *G*_MCGRP_. This outcome does not hold for the MO‐P‐MCGRP. Instead, it requires a multi‐graph, in which multiple arcs can exhibit the same orientation between two nodes.

To guarantee that the non‐dominated solutions on the multi‐graph are also non‐dominated on the original graph, all non‐dominated paths between two POIs for a given orientation must be included in the multi‐graph, which is easy to show:

1. When having all possible paths a non‐dominated solution on the multi‐graph is non‐dominated on the original graph.
2. Exchanging a dominated path with a path that dominates it only improves a solution.

In this case, a path *p*_*ij*_ = {*v*_1_, …, *v*_*m*_} between POIs *i* and *j*, consisting of segments *v*_1_ to *v*_*m*_, dominates a path *q*_*ij*_ = {*w*_1_, …, *w*_*m*_}, if there exists at least one segment of *q*_*ij*_ that is not in *p*_*ij*_, and every segment of *p*_*ij*_ is in *q*_*ij*_.

## 4 METHODS

The preprocessing of the multi‐graph and the ALNS and the three multi‐objective frameworks (MDLS, ECH, EBSH) and a combination of them (EBSH*), which share the former two as basic components, are described in the following subsections. We decided to use an ALNS with similar mechanism as presented in [12]. The dominance of this method is shown on the SINTEF homepage [38], where benchmarks for several instances along with the respective publications are presented.

Note that in [45] slightly better results with an alternative solution representation are presented. This representation is used in the genetic algorithm by [46] and the iterated local search by [35]. These results are, however, not provided on the SINTEF homepage. According to [45], the approach relies on the solution representation and very efficient move evaluation tailored for the single‐objective problem. Since an adaption to the multi‐objective problem is not straightforward, and ALNS comes very close in terms of solution quality, we decided to stay with ALNS.

### 4.1 Basic component: Multi‐graph generation

For creating multi‐graphs, we reimplemented an algorithm described in [26] to determine the *k*‐shortest paths with a specified diversity. Note that other recent research on multi‐graphs, where non‐dominated solutions are created, does exist (e.g., [5, 25]). However, they focus on a VRP with time windows, where cost and traversal time are taken into account. This allows for far easier and stronger cuts based on domination during the paths generation. Furthermore, it should be noted that the road‐network of our real‐world instances contains more than 20 000 nodes and 50 000 edges and arcs. This is by far larger than the largest networks in [25], where 500 nodes and 744 edges and arcs are included. Besides the problem of computational complexity in producing large networks, additional paths cause a higher computational effort during the ALNS. Therefore, we decided to rather generate a low number of high quality paths; results in Section 5.3 indicate that this was already sufficient for even slightly larger instances, as they deteriorate for some indicators when using three paths.

For the individual multi‐graph segments between two nodes *s* and *t* with *k*‐diverse shortest paths, we start with generating the first shortest path via the Dijkstra algorithm [14]. From this path {*s*, *x*_1_, …, *x*_*n*_, *t*} we generate all partial, deviating parts without loops and enter them into a priority queue (see Algorithm 1, lines 1‐4). For this, first all partial parts *p*_*i*_ = {*s*, *x*_1_, …, *x*_*i*_} are generated. Then every partial path is extended from the end node *x*_*i*_ to every possible neighbor *n*_*j*_(*x*_*i*_), except to the former successor. The resulting sets of deviating partial paths could be described as *P*(*p*_*i*_) = {{*s*, *x*_1_, …, *x*_*i*_, *n*_*j*_(*x*_*i*_)}| *n*_*j*_(*x*_*i*_) ≠ *x*_*i* + 1_}. Thereafter, we enter a loop for extending the most promising partial path in the priority queue until we have either generated the remaining *k* − 1 paths or there are no paths to extend. The partial paths are extended in the same manner as above, unless they do not have a successor. Obviously, paths that create loops ({*s*, …, *x*_*i*_, …, *x*_*i*_, …}) or that are already dominated by other paths are removed (see Algorithm 1, lines 5‐13).

To determine the most promising path, a lower bound for the minimum length required to satisfy the diversity is calculated. To measure similarity between two paths *S*_*i*_ and *S*_*j*_, we use the intersection length $\left(\frac{L\left(S_{i}\cap S_{j}\right)}{L\left(S_{i}\cup S_{j}\right)}\right)$, where we assume a maximum of 80%. This measure of similarity deviates from the one in the multi‐objective frameworks later used, but also strengthens the bounds. Thus, the path generation algorithm is accelerated significantly. Therefore, we would receive the lower bound (LB) for a partial path *p* due to an already accepted path *S*_*i*_ of.

(1) $$\frac{L\left(S_{i}\cap p\right)}{L\left(S_{i}\cup p\right)}\leq 0.8\Rightarrow \mathrm{LB}\left(p\right)\geq 2.25\times L\left(S_{i}\cap p\right)-L\left(S_{i}\right).$$

In addition to the lower bounds from already accepted paths, there is also the lower bound for the shortest distance from the current end node *x* of a partial path *p* to *t*, resulting in.

(2) LB(p)≥L(p)+L(x,t).

Algorithm 1 **Multi‐graph generation for *k* paths between *s* and *t***

1: *S*_1_←performDijkstraAlgorithm(*s*, *t*)

  2: *S* ← *S*_1_

  3: *PQ*←generateAllPartialPaths(*S*_1_)

  4: *PQ*←filterLoopingPathsAndSetLowerBounds(*PQ*, *S*)

  5: **while** !*PQ*. *empty* ∧ *S*.*size* < *k* **do**

  6:      *NP*←extendMostPromisingPath(*PQ*)

  7:      *NP*←filterLoopingPathsAndSetLowerBounds(*NP*, *S*)

  8:      *PQ* ← *PQ* ∪ *NP*

  9:      **if** *S*_*S*. *size* + 1_←includesNewFullPathSatisfyingDiversity(*NP*) **then**

10:           *S* ← *S* ∪ *S*_*S*. *size* + 1_

11:           *PQ*←setLowerBounds(*PQ*, *S*)

12:      **end if**

13: **end while**

14: **return** *S*

### 4.2 Basic component: Adaptive large neighborhood search

Using destroy and repair operators (*d* and *r*), ALNS iteratively selects pairs to apply to the incumbent solution *s*. The selection is based on scores updated in regular intervals of size *ν*, assigned to the used operators on the basis of their performance. Finding a new best solution s^*^, a new incumbent solution *s*, or a previously unidentified solution are events that prompt the awarding of a score to the pair of operators. Following any updates, the old scores are replaced by new scores. The new solution *s*′ then undergoes an acceptance test via simulated annealing (SA) to determine, whether it is accepted as new incumbent solution or not. SA has a *temperature* and a *cooling* parameter. As long as the temperature is high, the likelihood of solutions being worse than the current one to be accepted is also high ‐ promoting diversification. Cooling indicates how much the temperature is lowered in each iteration. This process repeats until it reaches a given stopping criterion (see Algorithm 2, lines 1‐4, 8, 14‐20).

For the operators, we mostly follow [34]:

1. *Destroy Random*: Random POIs are removed.
2. *Destroy Worst*: The most beneficial removals are executed.
3. *Destroy Related*: A random POI *r* is selected and removed together with a number of POIs that are closest in distance to *r*.
4. *Destroy Small*: POIs with the smallest demands are removed.
5. *Destroy Route*: A route is randomly selected. All POIs included in this route are removed.
6. *Destroy Historical Pair*: A pair of POIs *ij* with the largest historical values is removed. The historical value of *ij* is the best objective value observed for a route that includes *ij*.
7. *Repair Random*: POIs are inserted into random positions.
8. *Repair Greedy*: POIs are sequentially inserted into the position that leads to the smallest increase in cost.
9. *Repair Regret*: POIs are sequentially inserted into the position with the highest opportunity cost.

For all operators except for 1, 5, and 7, we include noise factors *u*, which we randomly draw from an interval *U* = [1 − *ψ*, 1 + *ψ*]. The values used to select POIs are multiplied by 1 + *u*, which increases diversification, because operators 1 and 7 are only used if no new best solution has been found for several iterations.

For further diversification, we design the operators such that promising but infeasible solutions are generated as well. For this effort, capacity and vehicle violations are included but penalized. We adjust penalties during the search procedure, depending on the number of observed violations. The objective values used for determining whether a new solution should be accepted or not are as follows:

1. *s*.*objValue* = *s*. *cost* + *ω*_*cap*_ × *s*.*vCap* + *ω*_*route*_ × *s*.*vRoute*
2. *s*.*objValue* = *s*.*cons* + *ω*_*cap*_ × *s*.*vCap* + *ω*_*route*_ × *s*.*vRoute*
3. *s*.*objValue* = *s*.*cost* + *ω*_*cap*_ × *s*.*vCap* + *ω*_*route*_ × *s*.*vRoute* + *ω*_*cons*_ × *s*.*vCons*

The first and second values apply to the MDLS, and the third applies to the EBSH and ECH. Here, *ω*_*cap*_, *ω*_*route*_, and *ω*_*cons*_ are the penalties for each unit of capacity, route, and consistency violation, respectively.

The ALNS is strengthened by a variable neighborhood decent (VND), which initiates if an incumbent solution is within a given percentage of the best solution or better. Hence, the VND intensifies good solutions (see Algorithm 2, lines 5‐7). The following operators then follow the first fit procedure:

1. *Swap*: Two POIs are swapped.
2. *Or‐Opt Route*: Three paths of a route are removed and reconnected such that the POI sequences remain unchanged.
3. *2‐Opt Route*: Two paths of a route are removed and reconnected.
4. *2‐Opt Different Routes*: Two paths of different routes are removed and reconnected.
5. *Flip*: A path is removed and reinserted after the sequence being flipped.

If no new best solution results after a given number of iterations *ϕ*, operators 1 and 7 as well as VND are applied to the incumbent solution (see Algorithm 2, lines 9‐13).

Considering route inconsistency increases the amount of stored data for the ALNS significantly, because we store for every path a vector of components, whose average size typically increases with larger instances. Furthermore, the move evaluation becomes far more costly, since for every path to be changed, the changes in inconsistency due to its components (i.e., edges and arcs of the original MO‐P‐MCGRP) must be calculated.

Consequently, by using a multi‐graph, the average computation time of almost all operators is increased as well. In particular because of the increased number of operations that must be evaluated. For destroy operators (besides operator 2), the increase is negligible, since only disruptions in the routes after removing POIs must be fixed. This is done by finding the path, where the smallest increase in objective value is observed. For destroy operator 2, the best path to reconnect the multi‐graph has to be determined, every time removal benefits are observed. Complexity increases of repair operators 8 and 9, where calculation of insertion costs requires to determine the pair of paths yielding the smallest increase in objective value. These time consuming operations, quadruple on a multi‐graph with two paths, and increase by a factor of 9 if three paths are considered. We tried to reduce this increase by cutting‐off calculations once we observe that insertion costs will not decrease. However, computational complexity is still huge. The same applies to VND's neighborhoods, where, however, the impact is reduced due to VND's share of computation time being smaller.

Algorithm 2 **Adaptive‐Large‐Neighborhood‐Search**

1: {*s*′, *s*, s^*^}←generateInitialSolution()

  2: **while** *stoppingCriterionNotReached* **do**

  3:       {*d*, *r*}←selectDestroyAndRepairOperators(*D*, *R*, *scores*)

  4:       *s*′ ← *r*(*d*(*s*))

  5:       **if** *s*′. *objective* < *μ* × s^*^. *objective* **then**

  6:            *s*′←VND(*s*′)

  7:       **end if**

  8:       {*s*, s^*^, *scores*′, *f*}←acceptanceTest(*s*′, *s*, s^*^, *scores*′, *d*, *r*, *f*)

  9:       **if** *f* = *ϕ* **then**

10:            *s*←randomDestroyAndRepair(*s*)

11:            *s*←VND(*s*)

12:            *f* ← 0

13:       **end if**

14:       *n* ← *n* + 1

15:       **if** *n* = *ν* **then**

16:            {*scores*, *scores*′}←updateScores(*scores*, *scores*′)

17:            *n* ← 0

18:       **end if**

19: **end while**

20: **return** *s*

### 4.3 Solution approach: Adaptive large neighborhood search‐based multi‐directional local search

**FIGURE 6:**
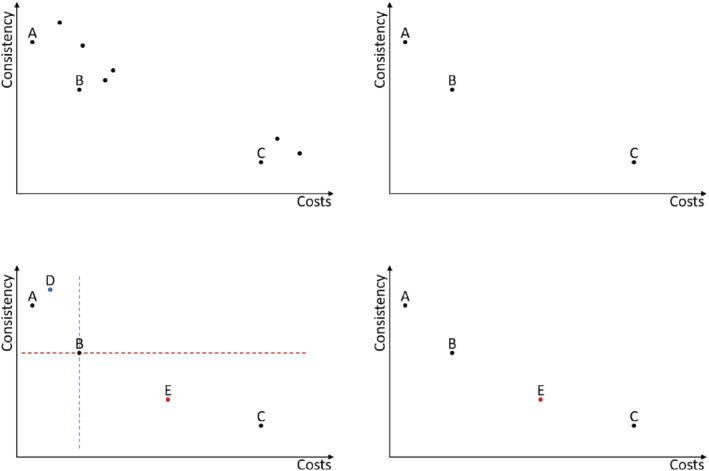
MDLS—initialization and first iteration. Top left: Initially generated solutions. Top right: Non‐dominated solutions from the initial set. Bottom left: Old solutions with two newly created solutions D and E from the randomly selected solution B. Bottom right: Non‐dominated solutions from the old and newly created solutions [Color figure can be viewed at wileyonlinelibrary.com]

For MDLS, introduced in [44], first a set *S* of non‐dominated solutions is created. This is followed by a random selection of a solution out of this set. A single‐objective heuristic for every objective is applied, such that a new solution per objective is created. Non‐dominated solutions are added to *S*. The procedure is repeated until a stopping criterion is met (see Algorithm 3 and Figure 6).

The ALNS described in Section 4.2 is used to optimize costs and inconsistency. To create the initial set of non‐dominated solutions, we use the proposed ALNS, such that we generate:

- 3 solutions using the single‐objective ALNS for costs.
- 1 solution by iteratively including previously non‐inserted POIs in each period, to fulfill capacity and inconsistency restrictions. If there is no such position available, then we insert the POI at a random position.
- 30 solutions using a random construction heuristic, such that the POIs are selected randomly and inserted into a random route that can meet capacity restrictions.

We then filter the non‐dominated solutions from this set of 34 solutions.

Algorithm 3 **Multi‐Directional‐Local‐Search**

1: *S*←generateInitialSolutions()

  2: **while** *stoppingCriterionNotReached* **do**

  3:       *s*←selectRandom(*S*)

  4:       **for** *objective* ∈ *Objectives* **do**

  5:            *s*′←ALNS_*objective*_(*s*)

  6:            *S* ← *S* ∪ *s*′

  7:       **end for**

  8:       *S*←filterNonDominatedSolutions(*S*)

  9: **end while**

10: **return** *S*

### 4.4 Solution approach: Adaptive large neighborhood search‐based *ϵ*‐constraint heuristic

The second multi‐objective solution approach for the MO‐P‐MCGRP is ECH, which is based on the *ϵ*‐constraint method [18]. First, it generates an initial solution by solving the single‐objective problem with the aim to minimize costs using ALNS (see Section 4.2). This solution's consistency (*secondObjective*) provides a constraint (*ϵ*‐constraint), when solving the single‐objective problem for the cost objective via ALNS, which has a time limit. This step repeats with the least consistent solution found so far (*S.last*) until no new solution fulfilling the *ϵ*‐constraint can be found or a time limit for the ECH has been reached (see Algorithm 4).

Algorithm 4 ***ϵ*‐Constraint‐Heuristic**

1: *S*←generateInitialSolution()

  2: *ϵ* ← *S*.*last*.*secondObjective*

  3: **do**

  4:       *newSolutionFound* ← *false*

  5:       *s*′← ALNS_*cost*_(*S*.*last*, *ϵ*)

  6:       **if** *s*′.*secondObjective* < *ϵ* **then**

  7:            *S*← filterNonDominatedSolutions(*S* ∪ *s*′)

  8:            *newSolutionFound* ← *true*

  9:            *ϵ* ← *S*.*last*.*secondObjective*

10:       **end if**

11: **while** *newSolutionFound*

12: **return** *S*

### 4.5 Solution approach: Adaptive large neighborhood search‐based *ϵ*‐box‐splitting‐heuristic

Similar to ECH, EBSH starts by solving the single‐objective problem for the first objective heuristically (see Section 4.2). After determining a box of the non‐dominated area, an iterative procedure starts, in which: (1) parameter *ϵ* is calculated on the largest box by splitting the box in two equal halves, (2) the single‐objective problem including the *ϵ*‐constraint gets solved via ALNS, and (3) the boxes and solutions are updated [29]. The algorithm stops if no boxes are left or a time limit is reached (see Algorithm 5).

Algorithm 5 ***ϵ*‐Box‐Splitting‐Heuristic**

1: *S*←generateInitialSolution()

  2: *B*←initializeBoxes()

  3: **while***stoppingCriterionNotReached* **do**

  4:       {*s*, *ϵ*}←findLargestBox()

  5:       *s*′←ALNS_*cost*_(*s*, *ϵ*)

  6:       **if** *s*′.*secondObjective* < *ϵ* **then**

  7:            {*S*, *B*}←filterNonDominatedSolutionsAndUpdateBoxes(*S*, *s*′, *B*)

  8:       **end if**

  9: **end while**

10: **return** *S*

**FIGURE 7:**
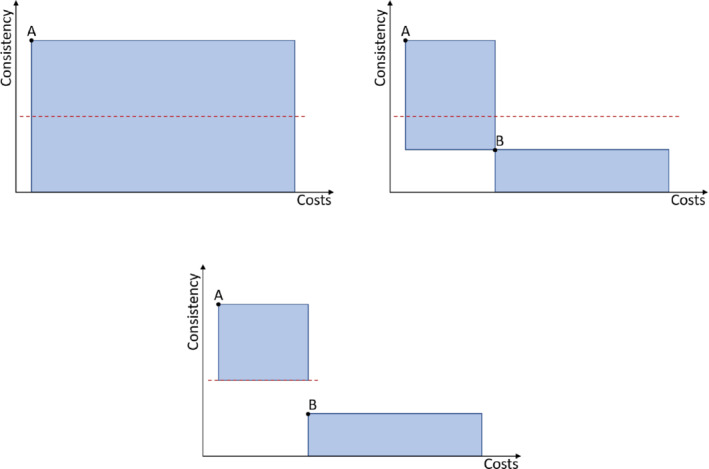
EBSH—Updating boxes. Top left: Box of solution A before splitting with blue line representing *ϵ*‐constraint. Top right: Boxes of solutions A and B after splitting. Bottom: Boxes of solutions A and B after splitting and removing examined solution space of A's box [Color figure can be viewed at wileyonlinelibrary.com]

To set the box sizes, this procedure assumes that both objectives are to be minimized. The first box uses the initial solution as one corner point and the lower/upper bound for the second/first objective. If a new solution emerges, all boxes are updated, and the dominated area is removed. If a box is split and no new solution emerges, the examined area can be removed as well, because there is thus a high probability that no Pareto solution is included (see Figure 7).

### 4.6 Solution approach: Adaptive large neighborhood search‐based two‐stage approach of *ϵ*‐box‐splitting‐heuristic and consistency‐ALNS

Our results for the real‐world instances in Section 5.3 indicated that EBSH was delivering on average the best results for all except one indicator (range covering). For this indicator, MDLS performed better. This was mostly due to MDLS finding more inconsistent solutions, and a few instances where MDLS found more cost‐efficient solutions. Therefore, we decided to combine EBSH with just the consistency‐ALNS of the MDLS to a two‐stage approach. This approach is denoted as EBSH* from now on. First, the EBSH is performed as before (see Algorithm 6, lines 1‐9). Then the least consistent solution and solutions being within a certain percentage of it are used as initial solutions *S*′ for the second stage (see Algorithm 6, line 10). For the second stage similarly to MDLS, solutions are picked at random and improved via the consistency‐ALNS until a stopping criterion is reached (see Algorithm 6, lines 11‐15), after which the non‐dominated solutions from both stages are determined and returned (see Algorithm 6, lines 16‐17).

Algorithm 6 *ϵ*‐Box‐Splitting‐Heuristic*

1: *S*←generateInitialSolution()

  2: *B*←initializeBoxes()

  3: **while** *stoppingCriterionNotReached* **do**

  4:       {*s*, *ϵ*}←findLargestBox(*S*, *B*)

  5:       *s*′←ALNS_*cost*_(*s*, *ϵ*)

  6:       **if** *s*′.*secondObjective* < *ϵ* **then**

  7:            {*S*, *B*}←filterNonDominatedSolutionsAndUpdateBoxes(*S*, *s*′, *B*)

  8:       **end if**

  9: **end while**

10: *S*′←filterLeastConsistentSolutions(*S*)

11: **while** *stoppingCriterionNotReached* **do**

12:       *s*←selectRandom(*S*′)

13:       *s*′←ALNS_*consistency*_(*s*)

14:       *S*′←filterNonDominatedSolutions(*S*′ ∪ *s*′).

15: **end while**

16: *S*←filterNonDominatedSolutions(*S* ∪ *S*′)

17: **return** *S*

## 5 COMPUTATIONAL STUDY

In the first part of the computational study, we apply the proposed ALNS to publicly available, standard MCGRP instances [1, 8, 36]. We show that the algorithms yield good results for single‐objective problems. In the second part, we focus on multi‐objective problems and apply MDLS, EBHS, and ECH to both artificial and real‐world instances. Tables 1 and  3 list the parameter settings for the single‐ and multi‐objective results; for the single‐objective case, they were set similar to ones found in the literature, whereas we tuned them via Irace [27] for the multi‐objective case.

**TABLE 1:** Parameters for single‐objective case without any tuning

Destroy and repair operators	2‐6 and 8‐9
Noise *ψ*	0.05
Starting violations for capacity *ω*_*cap*_, consistency *ω*_*cons*_, and routes *ω*_*route*_	{1000, 200, 1000}
Adaption for violations	Increase/decrease by 5%
Scores for ALNS	{10, 3, 1}
Cooling factor SA	0.999
Threshold of VND *μ*	1.05

### 5.1 Single‐objective results

We use several benchmarks to assess the performance of proposed ALNS for the single‐objective problem: MGGDB [8], BHW [1], CBMix [36], and DI‐NEARP [1]. These sets comprise a total of 205 instances, and except for the MGGDB, none of the instance types limits the number of vehicles.

The instances of MGGDB are rather small, ranging between 8 and 48 POIs, created from the 23 GDB instances. Six variations were created for each of those instances, by shifting the demand from required edges and arcs to adjacent nodes. The percentage of shifted required edges and arcs is indicated in the name by the middle part called *β* (e.g., mggdb_0.50_1 has 50% shifted). Therefore, instances with a higher *β* tend to have fewer POIs than those with a lower *β*. Furthermore, required nodes have higher demand than required edges and arcs, on average. Both BHW and CBMix represent medium size instance sets, such that CBMix instances range from 20 to 212 POIs, and BHW instances range from 20 to 410 POIs, though most are within the span of 110 to 240. The 24 DI‐NEARP instances are the largest, with 240, 422, 442, 447, 699, and 833 POIs, and 4 capacities for each. However, they do not include required arcs.

The ALNS was applied 15 times to every instance with a time limit after preprocessing of 1 hour, which is similar to [12, 45]. Table 2 lists the best, average, and worst percentage gap, relative to the best known results of the instances ($\frac{{\mathrm{res}}_{\text{ALNS}}-{\mathrm{res}}_{\text{bench}}}{{\mathrm{res}}_{\text{bench}}}$). The ALNS, sharing all the core components presented in [12], delivers results very close to the benchmarks. Thus, we deem our implementation well‐suited for the multi‐objective frameworks.

**TABLE 2:** ALNS results for single‐objective standard instances. We report average gaps

Instance type	Best Gap (%)	Avg. Gap (%)	Worst Gap (%)
MGGDB	0.00	0.00	0.00
BHW	0.00	1.57	4.74
CBMix	0.00	2.23	6.35
DI‐NEARP	1.84	3.05	4.60

### 5.2 Performance indicators for multi‐objective results

In accordance to [37], we select four indicators to evaluate the multi‐objective results, using a wide spectrum of criteria. The solutions were normalized to the range [1.0,2.0], where 1.0 and 2.0 are the best and worst results of the non‐dominated solutions, respectively. Hence, a normalized result of an individual instance can be larger than 2.0, if the result is worse than the worst solution regarding this objective of the non‐dominated solutions. Furthermore, since the set of all Pareto optimal solutions could not be generated for most instances ‐ even when using a MIP‐model ‐ we created reference sets *R* for the instances by taking all solutions from all runs and methods together and determining the non‐dominated solutions. For details on the selected performance indicators, see [37].

#### 5.2.1 Range covering

This indicator gives the closeness of an approximation set *A* to the minimum and maximum value for individual objectives. The closer the value to 1.0, the more the solutions reach the extreme points for the objectives.

#### 5.2.2 Hypervolume

The hypervolume indicator measures the weakly dominated area by a set *A*. We generally allow for normalized values above 2.0, so any larger values are set to 2.0, considering that (2.0,2.0) represents the nadir point with the worst objective values for the non‐dominated solutions. Accordingly, the results of the indicator must be within [0.0,1.0], and a higher values indicate better solutions than lower ones.

#### 5.2.3 Multiplicative unary *ϵ*

This indicator gives the multiplicator *ϵ* being applied to the objectives of solutions of reference set *R* such that approximation set *A* weakly dominates *R*. The smaller the value of this indicator, the better, with the best value being 1.0.

#### 5.2.4 R3

The R3 indicator calculates, for an approximation set *A* and a reference set *R*, the utility values given a set of weight vectors Λ. If the indicator is close to 0.0, set *A* yields similar utility to *R*.

(3) $$I_{R3}\left(A,R\right)=\frac{\sum_{\lambda \in \Lambda }\frac{u^{*}\left(\lambda ,R\right)-u^{*}\left(\lambda ,A\right)}{u^{*}\left(\lambda ,R\right)}}{|\Lambda |}$$

(4) $$u^{*}\left(\lambda ,A\right)=\max_{z\in A}\;u_{\lambda }\left(z\right)$$

To calculate utility, we use an augmented Tchebycheff function (5) with *ρ* and z^*^ fixed to 0.01 and 1.0, respectively.

(5) $$u_{\lambda }\left(z\right)=-\left(\max_{j\in 1,\ldots ,n}\lambda_{j},|,z_{j}^{*}-z_{j},|,+\rho \sum_{j\in 1,\ldots ,n},|,z_{j}^{*}-z_{j},|\right)$$

We assess the proposed multi‐objective frameworks on the basis of all performance indicators.

### 5.3 Multi‐objective results

We test the proposed multi‐objective frameworks with both the MGGDB instances and real‐world instances. We decided to only elaborate on the tests for the smaller artificial instances due to the heavy increase in problem complexity from the MCGRP to the MO‐P‐MCGRP. The real‐world instances cover 748 banks and automated teller machines (ATMs) located in the city of Vienna. To determine paths between these locations, we gathered street network data from an open source [11]. All instances can be downloaded from the BDA homepage [3].

Using these data, we created three paths between all banks and ATMs using an algorithm found in [26] and explained briefly in Section 4.2. Note that for the MO‐P‐MCGRP, including several shortest paths between POIs should increase solution quality, reflecting the second objective of route inconsistency. Since they, however, also cause complexity increase and larger computation times for the heuristics, it is important to limit the number of paths to a reasonable number.

MGGDB and real‐world instances do not considerably differ regarding the number of POIs. Paths of the real‐world instances, however, include far more edges and arcs. This results in more data handling for the ALNS, but also increases the amount of possible trade‐offs between costs and consistency, resulting in larger Pareto sets and more complexity for instances with a similar number of POIs.

Because no Pareto sets were available for the MGGDB and the proposed objectives, we used an approximation. This was created by taking all results from all runs and methods and determining a set of non‐dominated solutions. Before doing so, we tried to determine Pareto sets via the MIP‐model described in the Appendix. However, the complexity did not allow to find these within time limits of 72 hours for any but two instances.

For parameter tuning, we used Irace [27]. However, due to rather long runtimes of the multi‐objective frameworks, we decided to tune the frameworks' ALNSs by tuning the single‐objective ALNS for costs with a time limit of 1 minute per run and applying the results to them.

As test and training sets, real‐world instances of different sizes were taken. Considering the amount of parameters, we decided to perform two runs with a budget of 5000 each. First, a subset of parameters was tuned, then, while keeping the values for the first subset, the second subset was tuned. Table 3 lists the tuned parameters, the classification to subsets, their ranges, and the final setting after the tuning.

**TABLE 3:** Settings for parameter tuning in the multi‐objective case

Parameter	Subset	Range	Final setting
Noise *ψ*	1	[0; 0.5]	0.2
Capacity penalty *ω*_cap_	1	[1; 2000]	1674
Route penalty *ω*_route_	1	[1; 5000]	3445
Penalty increase	1	[1; 1.5]	1.49
Penalty decrease	1	[1; 1.5]	1.34
Cooling factor SA	1	[0.5; 0.9999]	0.9755
Threshold for VND *μ*	1	[1; 1.5]	1.09
Iterations for random destroy and repair *ϕ*	1	[50; 2500]	933
Score ALNS new best	2	[3; 100]	10
Score ALNS new incumbent	2	[2; 100]	3
Score ALNS new solution	2	[1; 100]	1
Update interval ALNS *ν*	2	[10; 200]	40

For EBSH and ECH, the time limit per iteration was set to 5 and 10 minutes, respectively. ECH's higher limit was caused by deeming it more important for ECH than EBSH to find a high‐quality solution within each iteration. In case of EBSH, a box and the solution space around it might be explored again later during the run, resulting in the chance to find formerly overlooked solutions. ECH, in contrast, cannot return to a search space above the current *ϵ* anymore. For MDLS, we applied time limits of 1, 5, 10, 30, 60, 120, and 300 seconds for the ALNS.

Across all four indicators, the best average was achieved with a time limit of 1 second for the ALNS. The loss in solution quality for larger time limits arises because the algorithm overlooks good solutions, while focusing on a single objective.

However, even with differing time limits for the ALNSs of the individual frameworks, we have equal global runtimes for all three methods.

#### 5.3.1 Results for artificial instances

In Table 4, we summarize results for the three basic multi‐objective frameworks across the MGGDB instances, with a time limit of 20 hours excluding preprocessing. The table also indicates whether the results are significant according to the Wilcoxon signed‐rank test [49]. A 99%‐significance is shown by dot (•) and prime (′), whereas a 99.9%‐significance is shown by diamond (◊) and star (⋆). A dot (•) and diamond (◊) indicate a significant improvement compared to the first of the other two entries in the subcolumn for the given indicator. A prime (′) and star (⋆) indicate a significant improvement compared to the second of the other two entries in the subcolumn for the given indicator. For example, in Table 4, the R3 indicator of −0.109 for the EBSH, marked by a diamond (◊) and prime (′), is with a significance of 99.9% different (better) than the first of the other two entries (MDLS) and with a significance of 99% different (better) than the second of the other two entries (ECH).

**TABLE 4:** Average results for the basic multi‐objective frameworks applied to MGGDB instances

		Methods		
Indicator	Objective	MDLS	ECH	EBSH
Range covering	↑	**0.932** ^**◊**⋆^	*0.836*	0.835
Hypervolume	↑	0.217	*0.648* ^**◊**^	**0.650** ^**◊**⋆^
Multiplicative unary *ϵ*	↓	1.528	*1.134* ^**◊**^	**1.132** ^**◊**^
R3	0	−1.034	*−0.113* ^**◊**^	**−0.109** ^**◊**^′

*Note*: Average indicator values are reported. Column *Objective* indicates whether the indicator aims for high values (↑), low values (↓), or closeness to 0. Best and second best values per indicator are displayed in bold and italic numbers, respectively. •, ◊, ′, ⋆ indicate significance according to the Wilcoxon signed‐rank test.

For three of the four indicators, MDLS is outperformed by ECH and EBSH. However, MDLS shows very good solution quality if we apply the range covering indicator, because ECH and EBSH have problems finding inconsistent solutions. When just comparing ECH and EBSH, EBSH delivers better results for three of the four indicators, though the results in all cases are rather close. Furthermore, the Wilcoxon signed‐rank test does not show a significant difference in the range covering and multiplicative unary *ϵ* indicators. These results for the artificial instances indicate that the ALNS‐based EBSH is the method of choice for the newly introduced problem. Except for cases where range covering is of a far greater importance.

#### 5.3.2 Results for real‐world instances

Tables 5, 6, 7 display the average results for the multi‐objective frameworks applied to real‐world instances with a time limit of 20 hours, excluding preprocessing. Again, we perform five iterations per framework and instance. For the real‐world instances, we distinguish between a simple‐graph and a multi‐graph setting. For the latter, we generate both the two and three shortest paths with maximum similarity of 80% (as explained previously). In Table 5 we compare MDLS, ECH, and EBSH against each other, while we compare MDLS and EBSH with EBSH*—a combination of the former two—in Table 7. Table 6 compares the effects of using a multi‐graph for all four frameworks.

**TABLE 5:** Average results for the multi‐objective frameworks applied to the real‐world instances, where 10, 20, or 30 POIs must be visited

			1 path			2 paths			3 paths		
Size	Indicator	Obj.	MDLS	ECH	EBSH	MDLS	ECH	EBSH	MDLS	ECH	EBSH
10	Range cov.	↑	**0.76** ^**•**^	0.67	*0.72*	*0.85*	0.84	**0.89**	*0.9*	0.82	**0.94**
	Hypervol.	↑	0.38	*0.51*	**0.52** ^**◊**^	0.34	*0.6* ^**◊**^	**0.63** ^**◊**⋆^	0.26	*0.67* ^**◊**^	**0.69** ^**◊**^
	Multipl. Unary *ϵ*	↓	*1.33*	1.34	**1.3**	1.33	*1.22* ^**•**^	**1.16** ^**◊**⋆^	1.38	*1.12* ^**◊**^	**1.11** ^**◊**^
	R3	0	*−1.06*	−1.15	**−0.93**	−0.85	*−0.36* ^**◊**^	**−0.27** ^**◊**⋆^	−0.89	*−0.16* ^**◊**^	**−0.05** ^**◊**⋆^
20	Range cov.	↑	**0.78** ^**◊**^	0.62	*0.63*	**0.87** ^**◊**^	0.68	*0.8*′	**0.85** ^**◊**^	0.7	*0.75*
	Hypervol.	↑	0.37	*0.57* ^**◊**^	**0.61** ^**◊**⋆^	0.32	*0.58* ^**◊**^	**0.63** ^**◊**⋆^	0.3	*0.57* ^**◊**^	**0.62** ^**◊**⋆^
	Multipl. Unary *ϵ*	↓	1.34	*1.3*	**1.27**	1.36	*1.27* ^**◊**^	**1.21** ^**◊**^	1.38	*1.26*	**1.22** ^**◊**^
	R3	0	−0.85	*−0.80*	**−0.64**	−0.70	*−0.68*	**−0.44**′	−0.77	*−0.67*	**−0.49** ^⋆^
30	Range cov.	↑	**0.82** ^**◊**⋆^	*0.63*	0.6	**0.87** ^**◊**^	0.66	*0.78*′	**0.85** ^**◊**^	0.65	*0.78*′
	Hypervol.	↑	0.28	*0.5* ^**◊**^	**0.58** ^**◊**⋆^	0.32	*0.49* ^**◊**^	**0.54** ^**◊**^	0.29	*0.48* ^**◊**^	**0.53** ^**◊**^′
	Multipl. Unary *ϵ*	↓	1.38	*1.33*	**1.27** ^⋆^	1.36	*1.31*	**1.26** ^**◊•**^	1.37	*1.32*	**1.27** ^**◊**^
	R3	0	*−0.88*	−0.95	**−0.64** ^**◊**⋆^	**−0.68** ^**◊**^	−0.92	*−0.70* ^⋆^	**−0.69** ^**◊**^	−0.99	*−0.73* ^⋆^

*Note*: Average indicator values are reported. Column *Obj*. indicates whether the indicator aims for high values (↑), low values (↓), or closeness to 0. Best and second best values per indicator are displayed in bold and italic numbers, respectively. •, ◊, ′, ⋆ indicate significance according to the Wilcoxon signed‐rank test.

**TABLE 6:** Average results for the multi‐objective frameworks applied to the real‐world instances, where 10, 20, or 30 POIs must be visited

			10 POIs			20 POIs			30 POIs		
Method	Indicator	Obj.	1 path	2 paths	3 paths	1 path	2 paths	3 paths	1 path	2 paths	3 paths
MDLS	Range cov.	↑	0.76	*0.85* ^•^	**0.9** ^◊^	0.78	**0.87** ^•^	*0.85* ^•^	0.82	**0.87**	*0.85*
	Hypervol.	↑	**0.38**	*0.34*	0.26	**0.37**	*0.32*	0.3	0.28	**0.32**	*0.29*
	Multipl. Unary *ϵ*	↓	**1.33**	*1.33*	1.38	**1.34**	*1.36*	1.38	1.38	**1.36**	*1.37*
	R3	0	−1.06	**−0.85** ^•^	*−0.89*	−0.85	**−0.7**	*−0.77*	−0.88	**−0.68** ^•^	*−0.69*
ECH	Range cov.	↑	0.67	**0.84** ^◊^	*0.82* ^◊^	0.62	*0.68*	**0.7**	0.63	**0.66**	*0.65*
	Hypervol.	↑	0.51	*0.6* ^◊^	**0.67** ^**◊**^ **′**	0.57	**0.58**	*0.57*	**0.5**	*0.49*	0.48
	Multipl. Unary *ϵ*	↓	1.34	*1.22* ^◊^	**1.12** ^**◊**^ **′**	1.3	*1.27*	**1.26**	1.33	**1.31**	*1.32*
	R3	0	−1.15	*−0.36* ^◊^	**−0.16** ^**◊**⋆^	−0.8	*−0.68*	**−0.67**	*−0.95*	**−0.92**	−0.99
EBSH	Range cov.	↑	0.72	*0.89* ^◊^	**0.94** ^**◊**⋆^	0.63	**0.8** ^•^	*0.75*	0.6	*0.78* ^◊^	**0.78** ^◊^
	Hypervol.	↑	0.52	*0.63* ^◊^	**0.69** ^◊^	0.61	**0.63** ^•^	*0.62*	**0.58** ^⋆^	*0.54*	0.53
	Multipl. Unary *ϵ*	↓	1.3	*1.16* ^◊^	**1.11** ^◊^	1.27	**1.21** ^◊^	*1.22*	1.27	**1.26**	*1.27*
	R3	0	−0.93	*−0.27* ^◊^	**−0.05** ^**◊**^ **′**	−0.64	**−0.44** ^◊^	*−0.49*	**−0.64**	*−0.7*	−0.73
EBSH*	Range cov.	↑	0.73	*0.92* ^◊^	**0.95** ^◊^	0.7	*0.83* ^•^	**0.93** ^**◊**⋆^	0.73	*0.86* ^•^	**0.91** ^◊^
	Hypervol.	↑	0.52	*0.62* ^◊^	**0.65** ^**◊**^ **′**	*0.63*	**0.64** ^⋆^	0.61	*0.57*′	**0.58** ^⋆^	0.53
	Multipl. Unary *ϵ*	↓	1.29	*1.15* ^◊^	**1.13** ^◊^	1.21	**1.15**	*1.15*	1.21	**1.15**	*1.19*
	R3	0	−0.91	*−0.25* ^◊^	**−0.1** ^**◊**⋆^	−0.47	*−0.29* ^•^	**−0.28** ^•^	−0.47	**−0.33**	*−0.41*

*Note*: Average indicator values are reported. Column *Obj*. indicates whether the indicator aims for high values (↑), low values (↓), or closeness to 0. Best and second best values per indicator are displayed in bold and italic numbers, respectively. •, ◊, ′, ⋆ indicate significance according to the Wilcoxon signed‐rank test.

**TABLE 7:** Average results for the multi‐objective frameworks applied to the real‐world instances, where 10, 20, or 30 POIs must be visited

			1 path			2 paths			3 paths		
Size	Indicator	Obj.	MDLS	EBSH	EBSH*	MDLS	EBSH	EBSH*	MDLS	EBSH	EBSH*
10	Range cov.	↑	**0.76**	0.72	*0.73*	0.85	*0.89*	**0.92**	0.9	*0.94*	**0.95**
	Hypervol.	↑	0.38	*0.52* ^◊^	**0.52** ^◊^	0.34	**0.63** ^◊^	*0.62* ^◊^	0.26	**0.69** ^◊^	*0.65* ^◊^
	Multipl. Unary *ϵ*	↓	1.33	*1.3*	**1.29**	1.33	*1.16* ^◊^	**1.15** ^◊^	1.38	**1.11** ^◊^	*1.13* ^◊^
	R3	0	−1.06	*−0.93*	**−0.91**	−0.85	*−0.27* ^◊^	**−0.25** ^◊^	−0.89	**−0.05** ^◊^	*−0.1* ^◊^
20	Range cov.	↑	**0.78**	0.63	*0.7*	**0.87**	0.8	*0.83*	*0.85*	0.75	**0.93** ^⋆^
	Hypervol.	↑	0.37	*0.61* ^◊^	**0.63** ^◊^	0.32	*0.63* ^◊^	**0.64** ^◊^	0.3	**0.62** ^◊^	*0.61* ^◊^
	Multipl. Unary *ϵ*	↓	1.34	*1.27*	**1.21** ^**◊**^ **′**	1.36	*1.21* ^◊^	**1.15** ^**◊**⋆^	1.38	*1.22* ^◊^	**1.15** ^**◊**⋆^
	R3	0	−0.85	*−0.64*	**−0.47** ^**◊**⋆^	−0.7	*−0.44*	**−0.29** ^**◊**⋆^	−0.77	*−0.49*	**−0.28** ^**◊**⋆^
30	Range cov.	↑	**0.82** ^◊^	0.6	*0.73*′	**0.87**	0.78	*0.86* ^⋆^	*0.85*	0.78	**0.91** ^⋆^
	Hypervol.	↑	0.28	**0.58** ^◊^	*0.57* ^◊^	0.32	*0.54* ^◊^	**0.58** ^◊^	0.29	*0.53* ^◊^	**0.53** ^◊^
	Multipl. Unary *ϵ*	↓	1.38	*1.27*	**1.21** ^**◊**^ **′**	1.36	*1.26* ^◊^	**1.15** ^**◊**⋆^	1.37	*1.27* ^◊^	**1.19** ^◊^
	R3	0	−0.88	*−0.64* ^◊^	**−0.47** ^**◊**⋆^	*−0.68*	−0.7	**−0.33** ^**◊**⋆^	*−0.69*	−0.73	**−0.41** ^**◊**⋆^

*Note*: Average indicator values are reported. Column *Obj*. indicates whether the indicator aims for high values (↑), low values (↓), or closeness to 0. Best and second best values per indicator are displayed in bold and italic numbers, respectively. •, ◊, ′, ⋆ indicate significance according to the Wilcoxon signed‐rank test.

The results for real‐world instances keep the trend of ECH and EBSH being better regarding the hypervolume, multiplicative unary *ϵ*, and, except for larger instances, R3 indicator, while being worse regarding the range covering indicator, except for the smaller instances with multiple paths. This indicates that ECH and EBSH explored far better the middle part of the Pareto frontier yielding far superior results for the hypervolume indicator and still reasonable better results for the multiplicative unary *ϵ* indicator. At the same time, however, MDLS was better at exploring the outside parts of the Pareto frontier due to having better results for the range covering indicators. This is likely also the cause for the R3 indicator being less in favor of ECH and EBSH, once they do not achieve competitive results for the range covering indicator, due to reaching a high utility, when inconsistency is valued highly.

The outliers of MDLS not being the best for the range covering indicator when looking at the smallest instances and at the same time using the multi‐graph can be explained by ECH and EBSH being more capable of exploiting the benefits of the multi‐graph for the still simple, small instances due to the individual ALNS‐runs of ECH and EBSH having far longer runtime than MDLS's.

When looking beyond the small instances, it can be examined that the multi‐graph approach still increases the results for the range covering indicator to a certain degree. This is not too surprising due to the inherent, before already explained limit of the conversion and the inclusion of multiple paths allowing for more inconsistent solutions. When comparing the ECH and EBSH, EBSH improved far more for the medium and large instance, while being roughly equal for smaller ones. We explain this by the problem still being very simple for the smaller ones enabling the underlying ALNS for ECH and EBSH to converge against the same value within the given time limit, while not being able to do so for medium and larger ones. Our implementation of the ECH ends as soon as no new solution is found during an iteration, whereas the EBSH would continue with trying to split the largest remaining box. Therefore, our EBSH, when given a lot of computation time, often used more computation time in total allowing for more exploration. This furthermore explains the trend of EBSH being slightly better than ECH with the Wilcoxon signed‐rank test still showing high significance even for larger instances. There is almost no difference and especially no high significance between using two or three paths. We explain this by the fact that there is not enough potential consistency increase, when adding the third path. However, since MDLS still had far better results compared to ECH and EBSH, these latter two methods still miss potential improvements. This implies that the additional complexity of the third path negated the potential gains.

The effect on the hypervolume, multiplicative unary *ϵ*, and R3 indicators seems even more mixed. When looking at ECH and EBSH, having multiple paths significantly improves the results for the smaller instances. Medium sizes ones, however, are only slightly improved and the Wilcoxon signed‐rank test does not show such a high significance anymore, while larger instances are even partially worsened; with even a significance of 99.9% for one specific comparison. This confirms that small instances can easily be exploited even with the increased solution space, while the additional complexity of more paths destroys the benefit of the Pareto set of the multi‐graph dominating the Pareto set of the simple‐graph. That this negative effect is more visible for the hypervolume, multiplicative unary *ϵ* likely stems from them being more complex indicators than range covering.

As the results indicate that the *ϵ*‐based methods and MDLS have different strengths, it might be reasonable to combine these methods. When comparing the results of MDLS and EBSH (since EBSH and ECH are very similar in their design, while EBSH performing better), we observe the following: almost all solutions of MDLS that are not of lower costs than EBSH's solution or less consistent than EBSH's least consistent solution are dominated by one or more solutions of EBSH. Secondly, there are on average very few solutions that are still more cost‐efficient than EBSH's (0.36) with an increasing but still small average for the larger instances (0.83). In comparison, the number of less consistent solutions is rather large with 2.16 and 3.80 on average for all instances and the larger instances, respectively. From these observations we conclude that the most promising combined method (denoted as EBSH^*^), is to (i) start with EBSH followed by (ii) short runs of MDLS's consistency‐ALNS with the least consistent solutions found as potential starting solutions. While there might also be more cost‐efficient solutions remaining, we consider it unlikely to find them within a short amount of time and only short ALNS runs. Instead it seems preferable to increase the time for generating the initial solution.

For the combined approach (EBSH*), we assign 19 hours of runtime for EBSH and 1 hour for MDLS's consistency‐ALNS. Hence, the total runtime is equal compared to the experiments of the individual approaches. Table 7 compares EBSH* to MDLS and EBSH. For the smallest instances, the results of EBSH and EBSH* are almost the same. Thus, the added search for inconsistent solutions does not yield any large improvements. However, for these instances we did not observe a large gap regarding range for any of the methods, anyway. For larger instances, no significant changes are apparent when comparing the hypervolume indiciator of EBSH and EBSH*, whereas significant improvements are revealed for the other three indicators. This seems to stem from the fact that they are more influenced by a few less consistent solutions. However, even for the larger instances when using one or two paths, EBSH* is on average still not significantly better than MDLS for the range covering indicator. We assume that the significance when having three paths is due to the closeness toward the maximum value for the objectives of EBSH*'s approximation sets, since MDLS's approximation sets are still closer toward the minimum value. Therefore, MDLS is still better at finding the extreme solutions for the single‐objective problems, with EBSH* closing the gap considerably.

### 5.4 Trade‐off between cost and inconsistency

Table 8 shows the average improvement in inconsistency (i.e., decrease in consistency) if costs increase by a certain percentage *p*, relative to the cheapest solution *s*_cheapest_ for an instance, which can be denoted as:

(6) $$\min_{s\in S}\left\{\frac{s.\text{cons}-s_{\text{cheapest}}.\text{cons}}{s_{\text{cheapest}}.\text{cons}},|,s.\text{cost}\leq s_{\text{cheapest}}.\text{cost}\times \left(1+p\right)\right\}.$$

**TABLE 8:** Trade‐off between cost increase and consistency decrease

	Consistency decrease		
Cost inc.	1 path	2 paths	3 paths
%	−10.58	−13.18	−13.69
%	−16.08	−17.80	−18.39
%	−21.93	−24.03	−25.22
%	−25.32	−28.56	−29.81
No limit (%)	−25.49	−29.60	−31.31

We only consider the non‐dominated solutions of the 13 real‐world instances. The first column shows the percentage increase, and of the lowest cost, while the remaining columns show the average reduction in consistency for the versions with 1 to 3 paths.

We observe that even a very small cost increase of 1% can lead to a considerable decrease in consistency, of at least 10%. Further cost increases lead to additional consistency decreases, though these weaker trade‐offs are weaker. Furthermore, the trade‐offs are more beneficial with multiple paths, and the maximum consistency reduction is reached with three paths.

## 6 CONCLUSION

We introduced the MO‐P‐MCGRP, which stems from vehicle routing problems for security guards and cash‐in‐transit operations. Objects and streets must be serviced or traversed such that cost and route inconsistency over multiple periods are minimized and maximized, respectively. We have formulated the MO‐P‐MCGRP mathematically and transformed it to an asymmetric CVRP. An ALNS‐based search procedure, developed and benchmarked on single‐objective MCGRP instances, was embedded into three multi‐objective solution frameworks (MDLS, ECH, and EBSH) to generate solutions for the MO‐P‐MCGRP. We assessed the performance of these frameworks with four indicators taken from the literature. For almost all instances, we found MDLS to be the best approach regarding the range covering indicator, while the *ϵ*‐based methods were on average better for the other indicators. When comparing these two methods, we can additionally examine EBSH being significantly better than ECH in many cases, without cases being the other way round. A combined approach of iteratively applying MDLS's consistency‐ALNS within the EBSH framework, did not show an effect on small instances, but slightly improved results for the larger instances. Furthermore, we observed that considering more than one shortest path between POIs might significantly increase solution quality. However, the additional computational effort of handling multi‐graphs for larger instances caused them to have less benefits or even worse results, hinting strongly at a simple‐graph being the method of choice for even larger or more complex instances. The trade‐off between cost and inconsistency revealed that the desired inconsistency increases significantly with just a small cost increase, and these trade‐offs grow even more beneficial when a multi‐graph is used. All real‐world instances are made publicly available in order to stimulate more research in this challenging and practically relevant area.

## APPENDIX: MATHEMATICAL MODEL 1

Besides using heuristics, we tried to use mixed integer programming to gain exact results; aiming to use them as benchmarks for the heuristics. However, due to the high complexity of the MO‐P‐MCGRP, the complete Pareto‐frontier could only be calculated for two of the smallest MGGDB instances within the time limit of 72 hours.

For the mathematical formulation of the transformed MO‐P‐MCGRP, let *N*_0_/*N*, *K*_*ij*_, *P*, and *L* denote the set of POIs with/without a depot, non‐dominated paths between POIs *i* and *j*, periods, and arcs and edges of the original MCGRP, respectively. For the paths, let $x_{\mathrm{ij}}^{\mathrm{kp}}$ be the decision variable indicating whether path *k* between POIs *i* and *j* has been used in period *p*, and let $c_{\mathrm{ij}}^{\mathrm{kp}}$ indicate the costs. Furthermore, $t_{\mathrm{ijk}}^{s}$ and $t_{\mathrm{ijk}}^{e}$ are binary values that denote the orientation of the POI at the start and end of the path, respectively. For example, $t_{\mathrm{ijk}}^{e}=1$ would indicate that POI *j* has orientation 1 after arriving there through $x_{\mathrm{ij}}^{\mathrm{kp}}$, and $t_{\mathrm{jik}}^{s}=0$ indicates that *j* is of orientation 0 after leaving it through $x_{\mathrm{ji}}^{\mathrm{kp}}$. Let *q*_*ip*_ be the remaining capacity after visiting POI *i* in period *p*, *d*_*ip*_ denote the demand, and *Q* represent the maximum capacity for a given set of vehicles *V*. For inconsistency in regard of paths, edges or arcs, decision variables *v*_*ij*_ and *v*_*l*_ are introduced. Via *v*_*ij*_ the number of times POI *i* is serviced directly before *j* except for the first time is determined, while *v*_*l*_ determines the number of times the edges and arcs of the original MCGRP are used beyond the first time. Finally, *w*_*l*_ is the weight of arc or edge *l*, and $b_{\mathrm{ij}}^{\mathrm{kl}}$ indicates whether *l* is part of path *k* between POIs *i* and *j*.

(A1) $$\min\sum_{i\in N_{0}}\sum_{j\in N_{0}}\sum_{k\in K_{\mathrm{ij}}}\sum_{p\in P}c_{\mathrm{ij}}^{\mathrm{kp}}x_{\mathrm{ij}}^{\mathrm{kp}}$$

(A2) $$\min\sum_{i\in N_{0}}\sum_{j\in N_{0}}v_{\mathrm{ij}}+\sum_{l\in L}v_{l}w_{l}$$

(A3) $$\sum_{i\in N_{0}}\sum_{k\in K_{\mathrm{ij}}}x_{\mathrm{ij}}^{\mathrm{kp}}=1\;\forall j\in N,p\in P$$

(A4) $$\sum_{i\in N_{0}}\sum_{k\in K_{\mathrm{ij}}}x_{\mathrm{ij}}^{\mathrm{kp}}\left(t_{\mathrm{ijk}}^{e}+1\right)=\sum_{i\in N_{0}}\sum_{k\in K_{\mathrm{ji}}}x_{\mathrm{ji}}^{\mathrm{kp}}\left(t_{\mathrm{jik}}^{s}+1\right)\;\forall j\in N_{0},p\in P$$

(A5) $$\sum_{k\in K_{\mathrm{ij}}}\sum_{p\in P}x_{\mathrm{ij}}^{\mathrm{kp}}\leq v_{\mathrm{ij}}+1\;\forall i\in N_{0},j\in N_{0}$$

(A6) $$\sum_{i\in N_{0}}\sum_{j\in N_{0}}\sum_{k\in K_{\mathrm{ij}}}\sum_{p\in P}x_{\mathrm{ij}}^{\mathrm{kp}}b_{\mathrm{ij}}^{\mathrm{kl}}\leq v_{l}+1\;\forall l\in L$$

(A7) $$\sum_{j\in N_{0}}\sum_{k\in K_{0j}}x_{0j}^{\mathrm{kp}}\leq V\;\forall p\in P$$

(A8) $$q_{\mathrm{ip}}+M-d_{\mathrm{jp}}-q_{\mathrm{jp}}-\sum_{k\in K_{\mathrm{ij}}}x_{\mathrm{ij}}^{\mathrm{kp}}M\geq 0\;\forall i\in N_{0},j\in N,p\in P$$

(A9) $$q_{0p}=Q\;\forall p\in P$$

The objectives, namely minimization of cost and consistency, which is equivalent to maximizing inconsistency, are formulated in (A1) and (A2), respectively. Constraints (A3) denote that every POI has to be serviced exactly once by requiring to have exactly one in‐going path. This works together with constraints (A4), since it causes the left side to be the orientation after arriving from *j*'s predecessor and the right side to be the orientation after leaving from *j* to its successor. By increasing all $t_{\mathrm{ijl}}^{e}$ and $t_{\mathrm{ijl}}^{a}$ by one, it furthermore guarantees that every node is left as well. The violations regarding the sequence of service are determined in (A5). This is done by adding up the number of times POI *j* was serviced directly after *i*. Similarly, constraints (A6) adds up the number of times edges and arcs of the original MCGRP were used to determine violations regarding street segments. The number of vehicles is restricted in constraints (A7) via the sum of outgoing arcs from the depot. We handle loading restrictions and also connectivity in constraints (A8), where the remaining capacity of the vehicle after servicing POI *j* is calculated, and constraints (A9), where the base capacity for the vehicles is set.
